# Acute exercise‐induced improvements in cognition: Role of cerebral blood flow and metabolism

**DOI:** 10.1113/EP092670

**Published:** 2025-11-19

**Authors:** Takeshi Hashimoto, Shigehiko Ogoh

**Affiliations:** ^1^ Faculty of Sport and Health Science Ritsumeikan University Kusatsu Japan; ^2^ Department of Biomedical Engineering Toyo University Asaka Japan

**Keywords:** glucose, individual differences, lactate, oxygen, regional cerebral blood flow

## Abstract

Physical activity is widely recognized for its ability to promote brain health, with acute exercise transiently enhancing cognition and long‐term training attenuating cognitive decline. However, the mechanisms underlying these benefits remain incompletely understood. Cerebral blood flow (CBF) has traditionally been considered central to exercise‐induced cognitive improvements, given the brain's dependence on a continuous supply of oxygen and glucose. Yet, accumulating evidence indicates that changes in global CBF alone cannot fully explain enhanced cognitive performance. Instead, regional CBF responses through neurovascular coupling, as well as cerebral metabolism – including oxygen extraction, glucose utilization and lactate uptake – are likely more critical determinants of brain function in response to exercise. Importantly, substantial individual differences exist in these responses. While some individuals experience robust cognitive gains from identical exercise regimens, others show little or no benefit. Emerging evidence suggests that variability in glucose tolerance, lactate dynamics and exercise capacity may underlie this heterogeneity, reflecting differences in metabolic responses and cerebrovascular regulation. For example, impaired glucose utilization might be linked to diminished exercise‐induced cognitive improvement, whereas lactate uptake appears to support high‐intensity exercise‐related gains. These findings highlight that the cognitive effects of exercise are not uniform, but rather influenced by individual physiological characteristics. This review therefore emphasizes the integrative regulation of CBF and metabolism as key factors mediating exercise‐induced cognitive improvements, while emphasizing the importance of inter‐individual variability. Understanding why some individuals benefit more than others is essential for adapting exercise prescriptions to maximize brain health across diverse populations.

## Abstract (highlights)

- **What is the topic of this review?**
  This review explores how in response to exercise cerebral blood flow (CBF) and metabolism interact to affect cognitive function.
- **What advances does it highlight?**
  The review emphasizes evidence showing exercise‐induced cognitive improvements cannot be explained solely by global CBF changes, but may be linked to cerebral metabolism, including oxygen extraction, glucose utilization and lactate uptake. It also highlights the need for studies on how CBF and cerebral metabolism are regulated during exercise and under conditions such as diabetes and stroke, while considering individual differences.

## 1 IMPACT OF CEREBRAL BLOOD FLOW AND METABOLISM ON BRAIN FUNCTION IN RESPONSE TO EXERCISE

Physical activity has been shown to be effective in preventing and delaying dementia (Erickson et al., 2011), while meta‐analytic evidence indicates that physical inactivity is a significant risk factor for Alzheimer's disease (Norton et al., 2014). Exercise, a subcategory of physical activity that is planned, structured, repetitive and aimed at the goal of improving fitness, is widely recognized for its cognitive benefits. Exercise training is recommended for older adults, including patients with Alzheimer's disease, as an intervention to prevent dementia (Ahlskog et al., 2011; López‐Ortiz et al., 2021; Tari et al., 2025). However, there is a critical need for comprehensive investigations to determine the optimal intensity, duration and frequency of exercise that most effectively enhance cognitive function across diverse populations. Furthermore, the development and rigorous validation of personalized exercise prescriptions, tailored to individual characteristics, are essential to establish interventions that are both widely accessible and maximally effective in promoting cognitive health (Tari et al., 2025). The effects of exercise on cognitive function, however, can vary depending on physiological factors influenced by exercise workload and modality. For example, Han et al. (2025) recently reported that different exercise modalities provide domain‐specific cognitive benefits in healthy older adults, suggesting targeted interventions: resistance training for global cognition, mind–body exercise for executive function, and aerobic exercise for memory. Notably, this relationship may differ depending on the target population, such as young adults or individuals with dementia. Therefore, to maximize the cognitive benefits of exercise for all individuals, it is essential to elucidate the mechanisms underlying these exercise‐induced effects.

When considering the underlying mechanisms, it is important to recognize a complicating factor: the transient activation of cognitive function induced by acute exercise (Ogoh, 2017; Ogoh et al., 2014c) does not necessarily share the same mechanisms as the sustained maintenance of high‐level cognitive function achieved through habitual exercise training, which may involve myriad processes, including long‐term structural and functional adaptations in the brain (Hashimoto et al., 2021). Nonetheless, it is reasonable to consider that repeated transient cognitive enhancements induced by acute bouts of exercise may accumulate over time to yield sustained benefits for cognitive function. Therefore, clarifying and systemically characterizing the mechanisms by which acute exercise influences cognitive function is a critical step toward establishing evidence‐based exercise training prescriptions (Dora et al., 2024; Hashimoto et al., 2021). Indeed, the degree to which executive function improved by acute exercise is associated with the cognitive improvement induced by chronic exercise training (Voss et al., 2020). However, numerous studies have reported complex and sometimes inconsistent findings regarding the cognitive effects of acute exercise. For example, a low exercise workload may fail to improve cognitive function even when the total work is comparable to that of an exercise regimen that does produce improvements (Saito et al., 2021; Washio et al., 2021). Moreover, there are substantial individual differences in these cognitive benefits: while some individuals experience improvements from the same exercise regimen, others do not (Kunimatsu et al., 2025). Although previous studies have attempted to clarify these mechanisms, they remain incompletely understood. This review aims to summarize current evidence on the mechanisms underlying exercise‐induced cognitive improvements, with a particular focus on emerging insights into cerebral metabolism and its interaction with cerebral blood flow (CBF) during exercise. However, it should be noted that the mechanisms underlying cognitive effects differ during and after exercise. For example, during exercise, activation of motor‐related brain regions may limit the recruitment of cognitive‐related brain regions (Dietrich, 2006; Moriarty et al., 2019). In contrast, these cognitive‐related brain regions may be more broadly and strongly activated following exercise (Dietrich, 2006; Moriarty et al., 2019).

## 2 CEREBRAL BLOOD FLOW IN EXERCISE‐INDUCED COGNITIVE IMPROVEMENT

Dementia often co‐occurs with stroke, and the prevalence of cognitive impairment after stroke is particularly high following recurrent strokes. Hennerici (2009) proposed hypotheses regarding the mechanisms of stroke‐induced dementia, suggesting that cognitive decline and the pathogenesis of dementia in stroke patients are more strongly influenced by the deterioration of pre‐existing cerebral and systemic vascular factors associated with stroke than by the stroke itself. Moreover, cerebrovascular dysfunction often precedes the onset of cognitive impairment, indicating its potential role in the development of dementia (Iadecola, 2004). In patients with Alzheimer's disease, dysregulation of cerebral circulation – including endothelium‐dependent responses (Iadecola et al., 1999), functional hyperaemia (Niwa et al., 2000), cerebral autoregulation (Niwa et al., 2002) and responses to vasoconstrictors (Niwa et al., 2001) – also occurs. Taken together, these findings strongly suggest that alterations in vascular risk factor‐induced attenuation of CBF or dysfunction in CBF regulation are closely associated with cognitive function. Against this background, increases in CBF are thought to enhance cognitive function. Thus, one possible mechanism underlying exercise‐induced improvements in cognitive function is an increase in CBF. This idea is supported by the fact that the brain requires a continuous supply of glucose and oxygen from the cerebral circulation to maintain its function, due to its relatively small energy reserves, and by classical findings showing that localized increases in CBF occur in response to neural activation (Roy & Sherrington, 1890).

Because exercise is known to elevate global cerebral blood flow (gCBF), many researchers have hypothesized that the cognitive benefits of exercise are mediated by this exercise‐induced increase in gCBF. Several studies (Ogoh et al., 2024, 2014c; Shirzad et al., 2022; Tari et al., 2020) have examined the effects of gCBF increases, both at rest and during exercise, on cognitive function. Shirzad et al. (2022) reported that both active and passive exercise increased gCBF and improved executive function post‐exercise compared with the control condition. However, the gCBF increase was greater in the active exercise condition than in the passive exercise condition, with corresponding larger improvements in cognitive performance observed following active condition. The same group (Tari et al., 2020) also showed that hypercapnia‐induced gCBF elevation enhanced executive function. These findings suggest that exercise‐related improvements in executive function are mediated by increased gCBF. However, these studies (Shirzad et al., 2022; Tari et al., 2020) have key limitations. The passive exercise protocol (Shirzad et al., 2022) reduced both gCBF and central command compared with the active condition, making it difficult to isolate the effect of gCBF on cognitive function. Another study (Tari et al., 2020) showed that exercise‐induced increases in gCBF improved executive function, but this effect was observed after, not during, exercise (i.e., without increased gCBF). Notably, although cognitive improvements occurred in both conditions (exercise and hypercapnia), the relationship between gCBF changes and cognitive performance after exercise differs from that under hypercapnia alone, suggesting that changes in gCBF alone cannot fully account for cognitive improvement. In addition, it is not possible to simply conclude that changes in oculomotor performance reflect changes in cognitive function. An increase in blood flow to the extraocular muscles controlling eye movements could directly affect their speed and accuracy, and sufficient oxygen supply to the retina or optic nerve may improve visual information processing and destabilize eye movements. Notably, physiological conditions immediately after endurance exercise are associated with dilated retinal vessels (Eijgen et al., 2025), suggesting that exercise can enhance eye movement performance independently of cognitive function. These possibilities should be verified by future research. Collectively, these studies could not determine whether gCBF directly enhances cognitive function during exercise.

Our recent study (Ogoh et al., 2024) addressed these limitations and examined the effects of both increases and decreases in gCBF on cognitive function. To directly assess cognition, we analysed event‐related potentials (ERPs) obtained from time‐locked electroencephalography (EEG), focusing on the P300 component, which directly reflects cerebral neural activity related to cognitive function. This approach allowed us to isolate cognitive function from exercise‐related outcomes for specific evaluation. The results of this study indicated that neither increases nor decreases in middle cerebral artery mean blood flow (MCA *V*_mean_) altered neural activity as measured by the P300. Thus, changes in gCBF, whether elevations or reductions, may not directly influence cognitive function. However, caution is warranted when interpreting changes in MCA *V*, as they may not accurately reflect changes in gCBF. These findings suggest that alterations in cognitive function under different physiological conditions cannot be explained solely by changes in gCBF. Our previous study (Ogoh et al., 2014c) manipulated gCBF using hypercapnic gas to test whether increased gCBF improves cognitive function during prolonged exercise. The results suggest that gCBF changes are unlikely to affect cognition, indicating that acute exercise‐induced cognitive improvements are more likely due to neural activation than global cerebral circulation. In conclusion, exercise‐induced improvements in cognitive function are not simply due to changes in gCBF. However, there may be thresholds or conditions under which gCBF influences cognitive function, as extreme alterations in gCBF – such as those occurring during stroke – can profoundly affect cognition. Further investigation is needed to clarify these issues.

## 3 GLOBAL AND REGIONAL CBF EFFECT ON COGNITIVE FUNCTION

It is undeniable that the brain requires a continuous supply of glucose and oxygen from the cerebral circulation to maintain its function, given its relatively small energy reserves. The close spatial and temporal relationship between neural activity and regional CBF (rCBF) is termed ‘neurovascular coupling’. During synaptic activity, a sudden increase in energy demand can lead to a relative shortage of oxygen and glucose in the brain. Therefore, cognitive function is thought to depend on an adequate increase in rCBF to support neural activity, even though gCBF itself may not directly affect cognitive function. Consequently, if gCBF influences the rCBF response induced by neurovascular coupling, it may indirectly affect cognitive function.

Global CBF is known to change under various conditions (Ogoh, 2015; Ogoh & Ainslie, 2009; Ogoh et al., 2014a, 2014b), and its response to physiological stress is important for maintaining brain metabolism and function (Bundo et al., 2002). Conversely, impaired interaction between neural activity and gCBF may contribute to brain dysfunction, as cerebrovascular dysfunction often precedes cognitive impairment (Iadecola, 2004). For example, in patients with dementia, cerebrovascular structure is altered, leading to a reduction in microvessels and degeneration of endothelial and smooth muscle cells (Farkas & Luiten, 2001). Additionally, resting gCBF is reduced, and the increase in rCBF in response to physiological stimuli is attenuated (Hock et al., 1997; Mentis et al., 1996; Warkentin & Passant, 1997). These findings suggest that regulation of gCBF can influence rCBF responses to neural activity, and as a result, may affect cognitive function even during exercise. However, three previous studies (Nowak‐Flück et al., 2018; Willie et al., 2011; Yamaguchi et al., 2014) demonstrated that exercise did not change neurovascular coupling despite increase in gCBF. Thus, exercise‐induced improvement in cognitive function may not be due to alterations in neurovascular coupling.

Indeed, cerebral neural activity requires an increase in rCBF, but this primarily serves to supply oxygen and glucose to the activated brain regions. Thus, another potential mechanism for exercise‐induced cognitive improvement is ‘cerebral metabolism’. Importantly, cerebral metabolism is closely linked to changes in rCBF; however, mismatches can occur between rCBF and the actual delivery of oxygen or glucose, and lactate as well given that glycolysis proceeds to lactate under fully aerobic conditions (Brooks, 2018). Therefore, in the following paragraph, we will examine the effects of oxygen, blood glucose and blood lactate supplementation on cerebral metabolism, independent of gCBF changes during exercise.

## 4 RELATIONSHIP BETWEEN CEREBRAL O2 UPTAKE AND EXERCISE‐INDUCED COGNITIVE IMPROVEMENT

Again, the association between cognitive function and gCBF is based on the premise that changes in gCBF reflect alterations in cerebral oxygen delivery. Actually, hypoxic conditions both acutely and chronically impair brain function (Wang et al., 2022). In our previous study, a gradual reduction in gCBF during prolonged exercise was observed due to hyperventilation‐induced reduction in $P_{aCO_{2}}$ (Ogoh et al., 2014c). Nevertheless, cognitive function remained unaffected or even improved throughout the exercise period, despite the apparent mismatch between reduced gCBF and increased cerebral metabolic demand, reflected by cardiovascular drift. Additionally, augmenting gCBF via hypercapnia did not yield improvements in cognitive performance. These observations suggest that changes in gCBF are not necessarily paralleled by proportional changes in cerebral oxygen delivery or metabolic rate to support cognitive function along with prolonged exercise. In other words, the brain is likely perfused sufficiently to meet its oxygen demands, as the cerebral arteriovenous oxygen content difference maintains when gCBF is reduced. Indeed, Dalsgaard et al. (2004) demonstrated that the arterial–venous difference (a‐vDiff) of oxygen (i.e., brain oxygen uptake) shows a significant increase in response to a hyperventilation‐induced decrease in gCBF (Sato et al., 2011) during exercise until exhaustion, suggesting that change in a‐vDiff of oxygen may compensate for gCBF. Therefore, the exercise‐induced gCBF reduction caused by hyperventilation may not be considered detrimental to global cerebral energy metabolism.

We measured oxygen a‐vDiff in response to repeated bout of high‐intensity interval exercise (HIIE), and found that both the first bout and a second bout of HIIE significantly increased oxygen a‐vDiff (Hashimoto et al., 2018). Interestingly, we found that both bouts of HIIE enhanced cognitive function; however, the improvement observed after the second bout was smaller than that after the first. This finding suggests that the HIIE‐induced increase in brain oxygen uptake during exercise is not directly associated with the magnitude of cognitive enhancement, even though cognitive function was assessed during the recovery period following each HIIE (Hashimoto et al., 2018). Nonetheless, it should be noted that the a‐vDiff of oxygen reflects global brain oxygen uptake and is a key determinants of cerebral metabolism (i.e., the O_2_/carbohydrate uptake ratio) (Ide et al., 2000).

However, since oxygen a‐vDiff only reflects global oxygen uptake, assessment of regional brain oxygenation during exercise is also warranted. Shen et al. (2024) demonstrated in a systematic review regarding functional near‐infrared spectroscopy (fNIRS) studies that exercise alters prefrontal cortex and motor cortex oxygenation, which is associated with improvements in higher‐order cognitive functions. fNIRS serves as an indirect measure of neural activity, by capturing local increases in CBF reflected in changes in oxygenated and deoxygenated haemoglobin. Thus, future research should focus on regional cerebral oxygen uptake during exercise and its relationship to cognitive responses.

## 5 BLOOD GLUCOSE REGARDING EXERCISE‐INDUCED COGNITIVE IMPROVEMENT

Because blood glucose is crucial for the brain's metabolic function, in addition to oxygen, the beneficial effects of glucose provision and utilization during exercise are likely related to increasing, or at least maintaining, the brain's energy substrate supply (Nybo & Secher, 2004). Consistent with this notion, Ruegsegger et al. (2023) investigated how cognitive improvement following acute HIIE differ according to glucose tolerance status (i.e., normal glucose tolerance and impaired glucose tolerance) in adults with obesity. They demonstrated that exercise‐induced cognitive improvement is positively associated with better glucose tolerance, independent of body weight and composition, highlighting the importance of individual glucose utilization capacity for exercise‐induced cognitive improvement.

Nybo and Secher conducted a series of insightful experiments examining neuromuscular activation and cerebral metabolic responses during and immediately after prolonged exercise, with or without glucose supplementation. In one study, Nybo (2003) recruited male endurance athletes who performed 3 h of cycling under both glucose‐supplemented and placebo conditions, followed by maximal knee extension tests to evaluate muscle strength and central nervous system (CNS) activation. The findings demonstrated that exercise‐induced hypoglycaemia impaired CNS activation and reduced muscle strength, whereas glucose supplementation maintained blood glucose levels and preserved CNS activation even after 3 h of moderate‐intensity endurance exercise. Interestingly, under the placebo condition, when blood glucose decline to the threshold at which cerebral glucose uptake begins to decline (i.e., approximately 3.5 mM), ratings of perceived exertion (RPE) increased compared with the glucose‐supplemented condition. Overall, these findings indicate that glucose supplementation preserves neuromuscular activation and suggest that, once blood glucose falls below a certain threshold, cerebral metabolism may contribute to an increased perception of fatigue.

Furthermore, Nybo et al. (2003) conducted a randomized crossover trial in which participants performed 3 h of cycling with and without glucose supplementation. They found that blood glucose levels and the cerebral metabolic rate of oxygen (CMRO_2_), an index of cerebral oxygen metabolism, were maintained under the supplementation condition. Here, CMRO_2_ was calculated as the a‐vDiff of oxygen multiplied by MCA *V*_mean_. In contrast, in the placebo trial, blood glucose declined from 5.2 to 2.9 mmol/L after 3 h, accompanied by an increase in RPE, which consequently limited cerebral glucose uptake and reduce CMRO_2_, and cognitive dysfunction may occur (Amiel, 1998; Nybo et al., 2003). These findings suggest that the regulation of blood glucose during exercise directly affects cerebral energy supply and the perception of fatigue. However, the mechanism by which carbohydrate supplementation led to a higher CMRO_2_ compared with the placebo condition remains unclear. One possible explanation is that carbohydrate supplementation helps maintain blood glucose levels, thereby sustaining glucose delivery to the brain and promoting oxygen consumption, which in turn may increase CMRO_2_.

The benefits of carbohydrate supplementation are evident in endurance events such as ultramarathons. Inamura et al. (2024) reported that higher‐ranking finishers ingested significantly more carbohydrates than lower‐ranking finishers and observed that declines in blood glucose during race segments occurred more frequently in lower‐ranking finishers. Consistent with this, Ishihara et al. (2020) demonstrated that blood glucose concentrations during ultramarathons are positively correlated with running speed across race segments. Interestingly, Cona et al. (2015) showed that when ultramarathon runners were divided into ‘fast’ and ‘slow’ groups, faster runners exhibited superior cognitive performance compared with the slower runners. Taken together, these findings suggest a potential link between carbohydrate regulation during prolonged exercise and cognitive function. In this regard, carbohydrate supplementation appears to facilitate carbohydrate utilization, contribute to the maintenance of running speed, and may also exert beneficial effects on cognitive performance.

In this regard, it is noteworthy that individual differences exist in blood glucose responses to exercise. For example, some individuals exhibit a decline in blood glucose during exercise following carbohydrate ingestion, whereas others do not show any decline (Jentjens & Jeukendrup, 2002; Kondo et al., 2019). We have also reported inter‐individual variability in glycaemic responses to repeated bouts of aerobic exercise (Tsukamoto et al., 2023). Importantly, Schwarck et al. (2019) demonstrated that, in response to acute moderate‐intensity or high‐intensity interval exercise, some individual ‘responders’ showed improvements in cognitive function, whereas other, ‘non‐responders’ did not. These findings suggest that future research should take individual differences in systemic and cerebral metabolic responses into account when evaluating cognitive performance requirements. Although the relationship between inter‐individual variability in glycaemic responses or glucose utilization capacity and exercise‐induced cognitive enhancement remains unclear, future studies should address this perspective.

## 6 BLOOD LACTATE REGARDING EXERCISE‐INDUCED COGNITIVE IMPROVEMENT

In the previous section, we discussed the positive effects of glucose metabolism and supplementation on cognitive and physical performance. The oxidation of glucose is considered the preferred energy source for the brain, and because glucose (glycogen) stores within neural tissue are limited, a steady and continuous supply is essential (Nybo & Secher, 2004). In contrast, during high‐intensity exercise, it is well worth noting that the brain takes up lactate as an energy substrate. Passive transport of lactate across the blood–brain barrier is facilitated by monocarboxylate transporters and is considered to depend on the lactate concentration gradient between blood and brain (Pierre & Pellerin, 2005). Consequently, the brain can utilize circulating lactate when arterial lactate concentrations rise substantially. Indeed, with increasing exercise intensity or the continuation of high‐intensity exercise, cerebral lactate uptake increases markedly compared with glucose uptake (Ide et al., 1999, 2000; van Hall et al., 2009).

**FIGURE 1:**
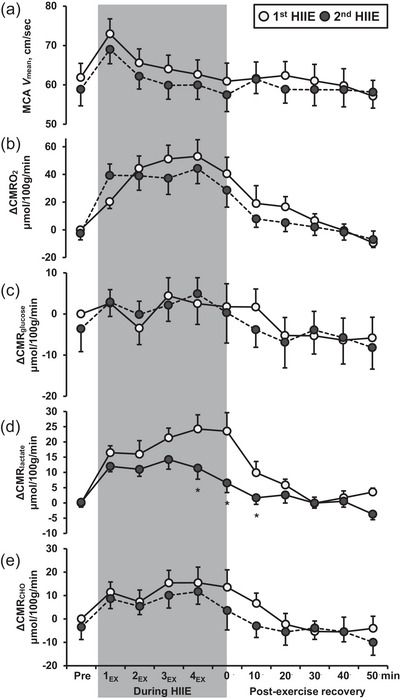
Changes in the MCA *V* and cerebral metabolic rates of each parameter in first and second HIIE conditions. Illustration of the changes in MCA *V*_mean_ (a), cerebral metabolic rates of oxygen (ΔCMRO_2_) (b), cerebral metabolic rates of glucose (ΔCMR_glucose_) (c), cerebral metabolic rates of lactate (ΔCMR_lactate_) (d), and cerebral metabolic rates of carbohydrate (ΔCMR_CHO_) (e) for the first (open circles) and second HIIE (filled circles). The values are expressed as the mean ± SEM. *P < 0.05 vs. first HIIE. Reproduced from Tsukamoto (2017) with permission.

We examined the relationship between cerebral metabolic responses and post‐exercise cognitive function when lactate production was attenuated due to muscle glycogen depletion by performing two repeated bouts of high‐intensity interval exercise (HIIE) (Hashimoto et al., 2018). Figure 1 illustrates the MCA *V*_mean_ during two repeated bouts of HIIE (Hashimoto et al., 2018), along with changes from rest in cerebral metabolic rate of oxygen (ΔCMRO_2_), cerebral metabolic rate of glucose (ΔCMRglucose), cerebral metabolic rate of lactate (ΔCMRlactate), and cerebral metabolic rate of carbohydrate (ΔCMRCHO). The latter indices were calculated at each time point as follows: [gCBF × a–vdiff Glucose], [gCBF × a–vdiff Lactate], and [gCBF × (a–vdiff Glucose + a–vdiff ½Lactate)], respectively. Changes in ΔCMRO_2_, an indicator of neural activity, increased during HIIE. Likewise, cerebral carbohydrate metabolism (ΔCMRCHO) was elevated. With respect to lactate, however, the second bout of HIIE induced lower lactate production than the first, resulting in a significant reduction in ΔCMRlactate that paralleled the decline in systemic blood lactate concentration (Hashimoto et al., 2018; van Hall et al., 2009). In contrast, ΔCMRO_2_, ΔCMRglucose and ΔCMRCHO did not differ between bouts, suggesting the maintenance of cerebral metabolism across repeated exercise. Notably, cognitive function decreased in proportion to the reduction in cerebral lactate uptake: compared with the first bout of HIIE, the second bout of HIIE elicited a lower blood lactate concentration, which was accompanied by a reduction in cerebral lactate uptake, and the enhancement of cognitive function observed after each HIIE session was diminished during the second bout relative to the first bout of HIIE (Hashimoto et al., 2018). In contrast, cerebral glucose uptake and ΔCMRglucose did not differ between the two bouts. These findings suggest that cerebral lactate uptake and CMRlactate play a pivotal role in mediating high‐intensity exercise‐induced improvements in cognitive function.

Recently, Green et al. (2024) evaluated the acute cerebral responses to moderate‐ and high‐intensity exercise in a total of 60 older adults, comprising 30 cognitively healthy individuals and 30 individuals with cognitive impairment. Their findings demonstrated that acute exercise reduced glucose metabolism across the entire grey matter, and this reduction was closely associated with the increase in systemic lactate concentration. These results suggest that lactate, whose levels rise during exercise, may serve as a primary energy substrate for the brain under such conditions (e.g., moderate‐to‐high intensity exercise). Given that in healthy humans glycolysis proceeds to lactate under fully aerobic conditions (Brooks, 2018), the aforementioned glucose supplementation may also facilitate lactate metabolism, thereby contributing to the preservation and improvement of both cognitive and physical functions. To further clarify this relationship, it will be necessary to experimentally manipulate lactate levels – for example, by employing a lactate clamp approach (Miller et al., 2002) – to examine the corresponding cerebral metabolic dynamics and cognitive outcomes.

Similarly to glucose response, individual differences in lactate response should also be considered. Kemppainen et al. (2005) directly assessed the effects of exercise intensity and exercise capacity on cerebral glucose uptake using positron emission tomography (PET). They found that regional glucose metabolic rate decreased across all measured cortical regions as exercise intensity increased, while lactate concentrations during exercise tended to show a negative correlation with cerebral glucose uptake. Notably, the reduction in glucose uptake in the dorsal anterior cingulate cortex – a region also implicated in cognitive function – was significantly greater in individuals with higher exercise capacity. Taken together with the previously discussed interindividual differences and the role of exercise capacity, these findings emphasize the need for further investigation into how exercise training alters cerebral metabolic dynamics and how such alterations may, in turn, influence cognitive function and physical functions on individual basis.

In summary, these previous studies suggest that blood glucose and exercise‐induced blood lactate are important factors influencing cognitive function, but this effect appears to be acute. It remains unclear whether these factors contribute to cognitive improvements associated with chronic exercise (Hashimoto et al., 2021). For example, Vestberg et al. (2012) demonstrated that football players exhibit higher cognitive function scores compared with the general population, and that ‘top‐level’ players possess even more advanced cognitive functions than lower‐level players. On the other hand, chronic hyperglycaemia, as seen in patients with diabetes, may affect cognitive function differently. In addition, caution should be exercised when extrapolating findings from acute studies in young healthy individuals, as discussed in this review, to chronic adaptation in clinical populations such as those with diabetes or stroke. Future studies should aim to integrate the regulation of CBF and metabolism in response to exercise and pathological or environmental challenges – such as exercise training, diabetes and stroke – while also taking individual differences into account.

## AUTHOR CONTRIBUTIONS

Takeshi Hashimoto and Shigehiko Ogoh conceived the review and prepared the original draft. Takeshi Hashimoto and Shigehiko Ogoh reviewed and edited the manuscript. Both authors have read and approved the final version of this manuscript and agree to be accountable for all aspects of the work in ensuring that questions related to the accuracy or integrity of any part of the work are appropriately investigated and resolved. All persons designated as authors qualify for authorship, and all those who qualify for authorship are listed.

## CONFLICT OF INTEREST

None declared.

## FUNDING INFORMATION

None declared.
